# Xylem transcription profiles indicate potential metabolic responses for economically relevant characteristics of *Eucalyptus* species

**DOI:** 10.1186/1471-2164-14-201

**Published:** 2013-03-22

**Authors:** Marcela Mendes Salazar, Leandro Costa Nascimento, Eduardo Leal Oliveira Camargo, Danieli Cristina Gonçalves, Jorge Lepikson Neto, Wesley Leoricy Marques, Paulo José Pereira Lima Teixeira, Piotr Mieczkowski, Jorge Maurício Costa Mondego, Marcelo Falsarella Carazzolle, Ana Carolina Deckmann, Gonçalo Amarante Guimarães Pereira

**Affiliations:** 1 Laboratório de Genômica e Expressão, Departamento de Genética Evolução e Bioagentes, Instituto de Biologia, Universidade Estadual de Campinas, São Paulo, Campinas, CEP: 13083-970, Brasil; 2 Department of Genetics, School of Medicine, University of North Carolina at Chapel Hill (UNC), Chapel Hill, NC, USA; 3 Centro de Pesquisa e Desenvolvimento em Recursos Genéticos vegetais, Instituto Agronômico de Campinas, São Paulo, Campinas, CEP: 13001-970, Brasil

**Keywords:** *Eucalyptus*, RNAseq, Transcriptome analysis, Secondary xylem

## Abstract

**Background:**

*Eucalyptus* is one of the most important sources of industrial cellulose. Three species of this botanical group are intensively used in breeding programs: *E. globulus, E. grandis* and *E. urophylla. E. globulus* is adapted to subtropical/temperate areas and is considered a source of high-quality cellulose; *E. grandis* grows rapidly and is adapted to tropical/subtropical climates; and *E. urophylla*, though less productive, is considered a source of genes related to robustness. Wood, or secondary xylem, results from cambium vascular differentiation and is mostly composed of cellulose, lignin and hemicelluloses. In this study, the xylem transcriptomes of the three *Eucalyptus* species were investigated in order to provide insights on the particularities presented by each of these species.

**Results:**

Data analysis showed that (1) most *Eucalyptus* genes are expressed in xylem; (2) most genes expressed in species-specific way constitutes genes with unknown functions and are interesting targets for future studies; (3) relevant differences were observed in the phenylpropanoid pathway: *E. grandis* xylem presents higher expression of genes involved in lignin formation whereas *E. urophylla* seems to deviates the pathway towards flavonoid formation; (4) stress-related genes are considerably more expressed in *E. urophylla*, suggesting that these genes may contribute to its robustness.

**Conclusions:**

The comparison of these three transcriptomes indicates the molecular signatures underlying some of their distinct wood characteristics. This information may contribute to the understanding of xylogenesis, thus increasing the potential of genetic engineering approaches aiming at the improvement of *Eucalyptus* forest plantations productivity.

## Background

Eucalypt is a ubiquitous tree in tropical and subtropical regions and is well known for its fast growth and adaptability. The *Eucalyptus* genus is composed of more than 600 species with an origin center based in Oceania [1]. Around 20 species are used in commercial plantations in more than 90 countries, most of them belonging to the subgenus *Symphyomyrtus*[2]. *Eucalyptus* wood is utilized in many ways, including for pulp, paper, civil construction, furniture and energy production [3]. In the near future, *Eucalyptus* may be an important source of second-generation biofuels and renewable chemicals. For all of these reasons, *Eucalyptus* has become the most planted forest genus in the world [4], which is a remarkable fact since these species are still in the early stages of domestication [2].

Three *Eucalyptus* species, *E. globulus, E. grandis* and *E. urophylla,* are generally the preferable genetic sources of the most desired breeding features [5]. *E. globulus* trees are well known for their superior wood properties for the paper and pulp industries and its lignin presents a high syringyl/guaiacyl ratio (S/G ratio), which is an important indicator of low recalcitrance during pretreatments [6]. In addition, the cellulose productivity of *E. globulus* is significantly higher than the average of other species; e.g., approximately 25% less wood is required to produce a ton of cellulose [7]. However, this species is not well adapted to tropical conditions [8] and is primarily cultivated in temperate countries such as Portugal, Spain and Uruguay.

In contrast, the wood of *E. urophylla* presents a high lignin content with a low S/G ratio and this species is very well adapted to high temperatures, humidity and drought, which are typical conditions of tropical areas. This species is widely used in breeding programs due to its superior resistance to adverse conditions, mainly to drought stress and diseases [9]. Amongst these three species, *E. grandis* exhibits the fastest growth and moderate resistance to disease and environmental extremes [10].

Although still poorly understood, it has been observed in some commercial forests in Brazil that the lignin and cellulose contents of *E. grandis* are intermediate between *E. globulus* and *E. urophylla*[11]. Furthermore, despite previous efforts to understand *Eucalyptus* wood formation, there is still a lack of knowledge regarding the major molecular differences of the xylems from these three species.

The comprehension of cell wall metabolism will lead to the improvement of *Eucalyptus* breeding programs and allow for the adoption of genetic strategies to enhance tree productivity, such as transgenic technology.

Wood is a result of secondary cell wall accumulation in xylem tissues, which occurs in four steps: (i) cell expansion, (ii) deposition of the secondary cell wall, (iii) lignification and (iv) programmed cell death [12,13]. Knowledge on the molecular mechanisms involved in cell wall biosynthesis and regulation has recently become available, and this area of research has been significantly enhanced by new “omics” technologies [14,15]. Since 2000, research consortiums have produced EST databases, such as Forest [16] and Genolyptus [7], Microarrays [17-19], SAGE [20] and DArT [21]. These databases are currently used to gain insights on the genetics of wood formation and various genes have been selected for the production of transgenic trees [22,23].

In recent years, new sequencing technologies have greatly improved our capacity of generating and assembling complex genomes as well as our ability of mapping and quantifying virtually all genes present in any given transcriptome with extreme precision [24,25]. The use of these technologies has allowed the sequencing and assembly of the *E. grandis* genome, and the *de novo* assembly of mRNAseq reads has provided a highly informative panel of *Eucalyptus* coding regions [26].

In this context, the aim of the present study was to use the RNAseq methodology to identify genes expressed in the xylems of *E. grandis*, *E. urophylla* and *E. globulus*, allowing the characterization of molecular signatures potentially associated with distinctive features related with their particular wood properties.

Results revealed that a relatively small number of genes (6518 or approx. 20% of all dataset) are differentially expressed in the developing xylem of these tree species, most of them with unknown function. Among those with a putative function assigned, we identified genes involved in cell wall synthesis and possibly associated to the diverse wood characteristics of *E. globulus, E. grandis* and *E. urophylla.*

## Results and discussion

### Xylem RNA sequencing

**Figure 1:**
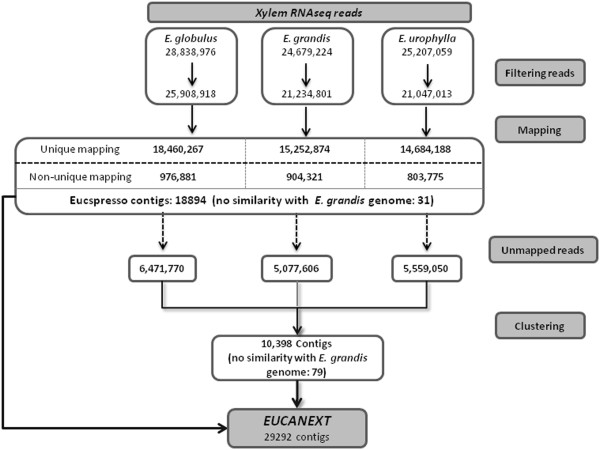
**Pipeline: Xylem transcriptome mapping and *de novo *assembly pipeline.** Reads were filtered to exclude ribosomal and low quality. Mapping were done against *Eucspresso* contigs being the majority of reads mapped in only one sequence in *Eucspresso* (unique mapping) while less than 1000000 reads for each species mapped in more than one *Eucspresso* sequence (non-unique mapping). Unmapped reads were clustered and formed 10.398 contigs. Together, *Eucspresso* and *De novo assembly* formed the 29.292 EUCANEXT contigs.

Developing xylem tissues were collected from 3-year-old individuals of *E. globulus*, *E. grandis* and *E. urophylla.* Each sample was used for the construction of RNAseq libraries, which were sequenced using an Illumina Genome Analyzer II_x_ sequencer. Approximately 78 million single-end reads with lengths of 36 bp (approximately 2.75 Gbp) were produced. Of this total, 28,838,976, 24,679,224 and 25,207,059 reads were derived from *E. globulus*, *E. grandis* and *E. urophylla*, respectively (Figure 1).

### Construction of a reference sequence database (*EUCANEXT*)

Xylem RNAseq reads of all three species were filtered to exclude ribosomal and low-quality sequences (see methods for more details). The filtered reads were then mapped against the “*Eucspresso”* database (http://eucspresso.bi.up.ac.za), which contains the complete gene catalog generated by Mizrachi and colleagues during the *de novo* assembly of the hybrid *E. grandis X E. urophylla urophylla* (termed ‘*urograndis’*) ESTs obtained from several tissues [26]. Although we performed this mapping against a database of the ‘urograndis’ hybrid, reads from *E. globulus* were mapped with a precision comparable to that of the other two species (see pipeline in Figure 1), indicating a high sequence conservation among their genomes.

Reads from the three species that did not map against the *Eucspresso* dataset (17,108,426 reads of the three species) were clustered together to produce a *de novo* assembly of 10,398 contigs (mean size: 407.75 bp; Additional file 1). Together, the *Eucspresso* contigs and the *de novo* assembly resulted in a database containing 29,292 contigs, which was named ***EUCANEXT*** (http://www.lge.ibi.unicamp.br/eucalyptusdb; Nascimento *et al.*, unpublished data)*.* Figure 1 shows the pipeline used for the construction of the *EUCANEXT* database (the distributions of the lengths of the contigs are shown in Additional file 2: Figure S1).

### Expression analysis

For gene expression analysis, the xylem RNAseq reads were aligned against the 29,292 contigs present in the *EUCANEXT* database, allowing the estimative of FPKM values (fragments per kilobase of exons per million of fragments mapped) for each contig in each of the three species (Additional file 2: Figure S2).

**Figure 2:**
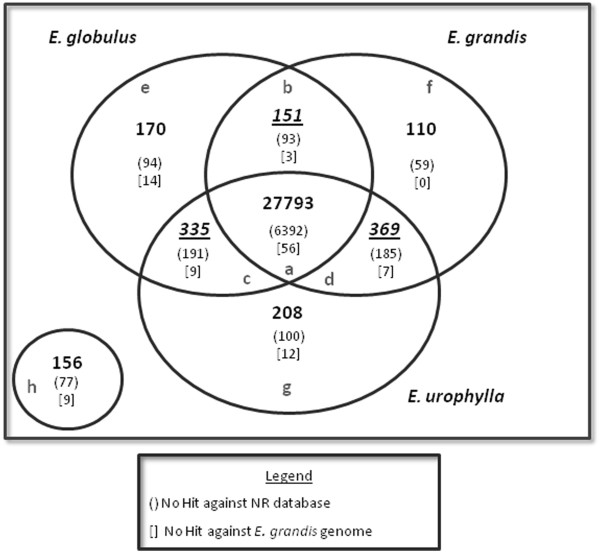
**Venn diagram showing the distribution of contigs between the *Eucalyptus *xylem libraries: distributions of contigs between species.** Bold numbers indicate contigs present in all species (**a**), contigs present in only two species (**b**, **c** and **d**), contigs present in only one species (**e**, **f** and **g**), and contigs not present in any species (**h**). The black box shows the legend for other numbers. (Presence of contigs defined as FPKM>0).

In order to identify the differentially expressed genes among the three species, the following pairwise comparisons were performed: *E. grandis* vs. *E. globulus*, *E. grandis* vs. *E. urophylla* and *E. globulus* vs. *E. urophylla.* From these comparisons, contigs were distributed in groups according to their expression profile in each pairwise comparison. Contigs with FPKM values equal to 0 were considered as non-expressed genes. Results are summarized by Venn diagram (Figure 2). A complete list of contigs is present in Additional file 3: Table S1.

In Figure 2, each subgroup is identified by a latin letter (‘a’, ‘b’, ‘c’…). Group ‘*a’* is composed by contigs with FPKM>0 in all three *Eucalyptus* species, i.e. genes considered expressed in all developing xylems. Groups ‘*b-d’* are composed by contigs with FPKM>0 in only two of the species studied, as following: *E. globulus* and *E. grandis (‘b’); E. globulus* and *E. urophylla; (‘c’); E. grandis* and *E. urophylla (‘d’).* Groups ‘e-g*’* are composed by contigs with FPKM>0 in only one species: *E. globulus (‘e’); E. grandis (‘f’); E. urophylla (‘g’).* Finally, group ‘*h’* is composed by contigs with FPKM=0 in all species, i.e. genes not expressed. A description of genes found in each group is provided in (Additional file 4: Doc file S1).

The 27,793 contigs comprising the group ‘*a*’ (Figure 2) represented 94.88% of the *EUCANEXT* contigs. The large number of genes common to the three transcriptomes suggests that the distinctive characteristics of each xylem may be derived from differences in the level of the expression of the same group of genes rather than the expression of a distinct set of genes. Also, it is possible to suggest that the cells of all three *Eucalyptus* species follow a common transcriptional program, which can be considered as a “xylem programming” and includes many genes involved in the production of precursors for secondary growth. Even in *E. globulus*, which is the most susceptible species to alterations in climatic conditions, the basic “xylem programming” presented essentially the same genes of the other two species.

The “a” group included a total of 5,175 genes differentially expressed in at least one species (Additional file 4: Figure S3). Thus, the majority of the genes (22,618) was not differentially expressed among the species (p-value > 0.01, fold change < 2; Additional file 4: Figure S3). This result was expected considering that the majority of the transcripts is involved in basal processes of the cellular metabolism. Therefore, these genes are essentially present at similar levels in equivalent tissues sampled from closely related species.

The group of contigs with FPKM>0 in only two species (groups ‘*b*’, ‘*c*’ and ‘*d*’, Figure 2) was composed of 855 contigs. Among the contigs that represented functionally annotated genes, we expected that, in each pairwise comparison, group of genes would reflect at least part of the phenotypic differences observed in the xylem of these *Eucalyptus* species. However, Gene Ontology (GO) analysis revealed that these genes are part of similar biological processes level 3 (Additional file 4: Figure S4 A).

The same pattern was verified in the groups of genes expressed in only one of the species (groups ‘*e*’, ‘*f*’ and ‘*g*’, Figure 2), in which contigs encoding known proteins (48%) were represented by genes displaying similar functions, as suggested by the Gene Ontology analysis (Additional file 4: Figure S4 B). The most important differences in gene expression for all groups are detailed in the next section, *Functional analysis.*

Of the 18,894 *Eucspresso* contigs present in the *EUCANEXT* database, only 156 (less than 1%) could not be detected in any of the three xylem RNAseq libraries (FPKM=0) (group ’*h*’, Figure 2). Because *Eucspresso* represents genes from several tissues, this finding indicates that the xylem tissues analyzed in this work presented an intense transcriptional activity, expressing >99% of all genes identified in the *Eucalyptus* transcriptome [26].

However, of the 156 undetected genes, 48% were derived from the “*urograndis*” hybrid xylem, as described by Mizrachi *et al.*[26]. This apparent contradiction may be reflecting some biologic event, as the presence of genes expressed as a result of the hybridization between *E. grandis* and *E. urophylla.* Alternatively, this result may just reflect the difference in the growth environment or plant age.

The analyses of the assembled contigs also included a comparison with the *E. grandis* genome database (http://phytozome.net/eucalyptus, Phytozome 8.0, with permission of Eucagen consorsium) to verify the quality of the dataset. For this analysis, *EUCANEXT* contigs were mapped against the *E. grandis* genome using BLASTn, resulting in the positive identifications of 29,106 contigs (99.36%) (e-value < 1e-10) (Figure 1). As expected, all 110 contigs that were presented only in *E. grandis* (Figure 2, group ‘f’) displayed similarity with the genome. However, some contigs in groups with FPKM>0 in *E. grandis* (Figure 2; groups ‘*a’, ‘b’* and ‘*d*’) did not map to the genome sequence, which may be a consequence of sequence gaps in the *E. grandis* genome assembly.

Interestingly, the proportion of contigs that did not map in the *E. grandis* genome for groups of contigs present in only one species (Figure 2; groups ‘*e*’ and ‘*g*’) was >2-fold higher when compared with other groups. The 14 contigs from *E. globulus* and 12 contigs from *E. urophylla* correspond to 8.23% and 5.76% of total genes for each group, respectively, while the proportion for other groups are: *group a*: 0.2%; *group b*: 1.98%; *group c*: 2.68%; *group d*: 1.89%. Despite these genes in groups ‘*e*’ and ‘*g*’ may also have occurred in *E. grandis* genome gaps, it suggests the presence of species-specific genes and are probably contributing to the distinct wood properties observed in these species.

The analysis of ‘*no hits’* genes expressed in xylem of the three *Eucalyptus* species (Figure 2, groups ‘*a*’ to ‘*g*’) demonstrated that they corresponded to nearly 21% of the non- differentially expressed genes present in all three transcriptomes, which further increases to approximately 60% among the genes present in only one or two libraries. Among all these *‘no hits’* genes*,* only 59% in average for each group in Figure 2, constitute ORFs ≥ 100 bp in length, a value usually used as an indicative of effective protein translation. These results may suggest that several species-specific or even tissue-specific *Eucalyptus* genes remain to be identified. It is expected that the number of genes with known functions will increase following the release of the complete genome annotation of *E. grandis*.

### Functional analysis

**Figure 3:**
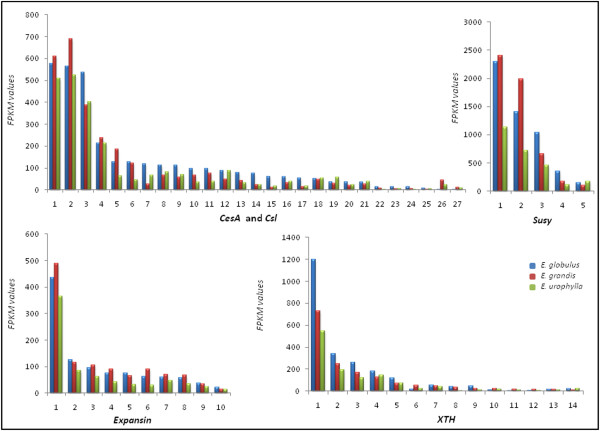
**Cell wall-related genes: the expression profiles of selected genes related to cell wall construction.** Numbers on the x-axis represent contigs listed in (Additional file 3: Table S2). The y-axis represents the FPKM values.

**Figure 4:**
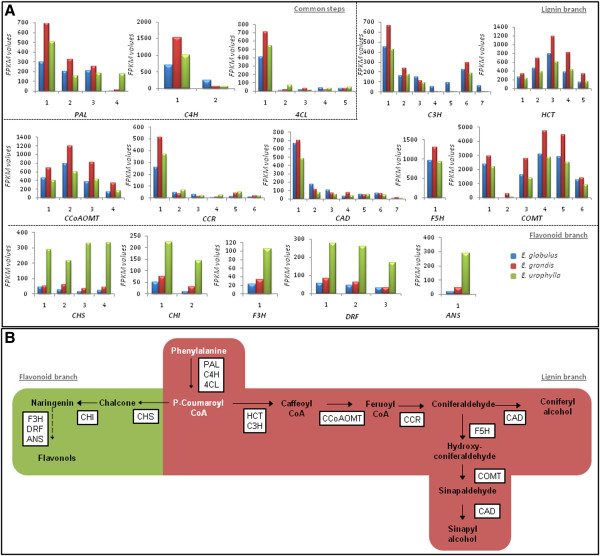
**Simplified phenylpropanoid pathway and related genes: A, the expression profiles of selected genes related to the phenylpropanoid pathway.** Numbers on the x-axis represent contigs listed in Additional file 3: Table S2. The y-axis represents the FPKM values. **B**, Common compounds shared by the lignin branch (right) and flavonoids branch (left) are depicted in white. Pathway genes are shown in white boxes (*PAL -* phenylalanine ammonium lyase; *C4H* - cinnamate-4-hydroxylase; *4CL* - 4-coumarate-CoA ligase; *HCT -* hydroxycinnamoyl-CoA:quinate shikimatep –hydroxycinnamoyltransferase; *C3H-*Coumaroyl-CoA 3-hydroxylase; *CCoAOMT* - caffeoyl-CoA 3-O-methyltransferase; *CCR* - cinnamoyl-CoA reductase; *F5H* - ferulate 5-hydroxylase; *COMT* - caffeic acid:5-hydroxyferulic acid O-methyltransferase; CAD - cinnamyl alcohol dehydrogenase; *CHS* - chalcone synthase; *CHI* - chalcone isomerase; *F3H* - flavonone 3-hydroxylase; *DFR* - dihydroflavonol 4-reductase; *ANS -* anthocyanidin synthase).The colored rectangles indicate genes that showed higher levels of expression in *E. grandis* (red) or in *E. urophylla* (green).

**Figure 5:**
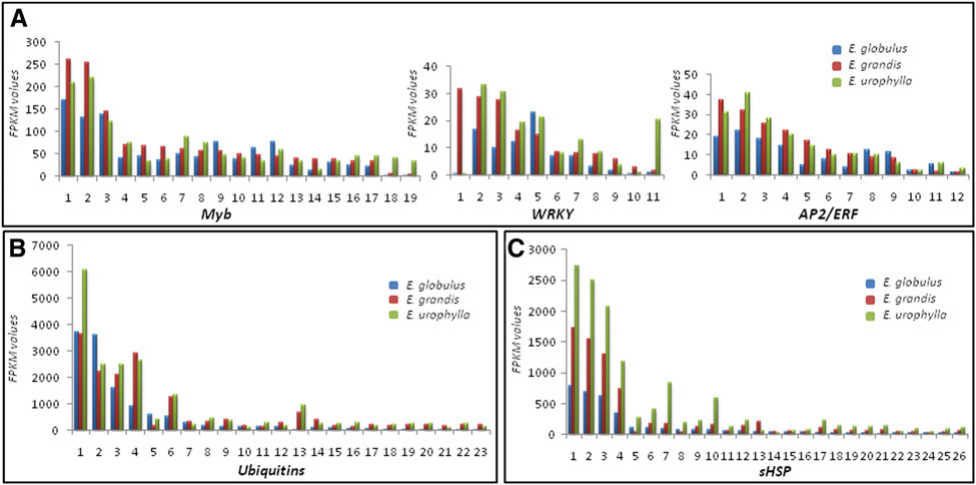
**Important selected genes: the expression profiles of selected genes related to: A, transcription factors; B,ubiquitins; and C, heat shock proteins.** The numbers on the x-axis represent the contigs listed in (Additional file 3: Table S3). The y-axis represents the FPKM values.

**Figure 6:**
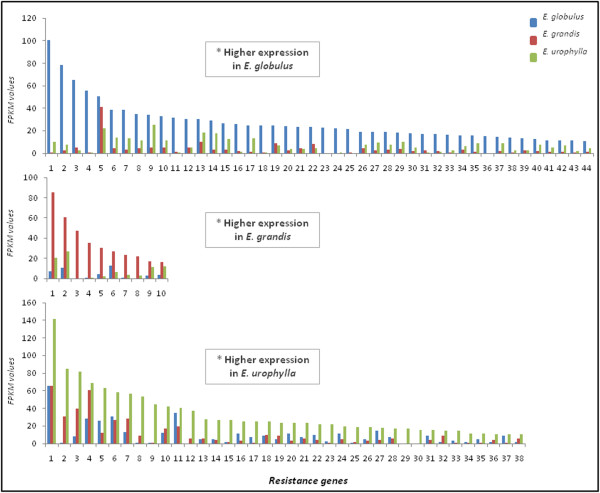
**Resistance-associated genes: the expression profiles of selected genes related to resistance.** Numbers on the x-axis represent the contigs listed in (Additional file 3: Table S3). The y-axis represents the FPKM values.

Several metabolic pathways involved in secondary wall formation and/or maintenance showed differential expression among species. They were analyzed in detail and compared among the three species. A great number of genes related to these pathways were found in our data (Additional file 3: Table S1), which indicates that large gene families encode each enzyme. In our discussion, only the members with highest expression were considered (Figures 3, 4, 5, 6 and Additional file 3: Table S2). In the following sections we show that gene expression of each family follows a general trend and can be observed through graphs.

The differences in gene expression found between *E. globulus* and other species should be analyzed in a more parsimonious way, as pointed out by the RT-qPCR analyses in Doc file S2 (Additional file 4). Correlations between RNAseq and RT-qPCR were stronger only in the comparison between *E. grandis* and *E. urophylla.* This result may indicate that *E. globulus* trees under stress conditions (such as tropical conditions) present heterogeneous molecular responses.

#### Secondary cell wall formation genes

Among the differentially expressed genes in at least one pairwise we identified several members of cell wall-related genes, as well as genes that directly or indirectly control wood formation. Despite detailed investigations are necessary to evaluate the relative contribution of each member, a general expression profile was observed for all members found, as described below.

Genes of the cellulose synthase superfamily (*CesA* – *cellulose synthase* and *Csl – cellulose synthase like*) and xyloglucan endotransglucosylase/hydrolase (*XTH*) family showed a lower level of expression in *E. urophylla* (contigs 1, 2, 5, 6, 10, 11, 13, 22, 24–27 for *CesA* and contigs 1–3, 7–9, 13 for *XTH*, Figure 3) and/or higher levels in *E. globulus* (contigs 3, 6, 7–11, 13–17, 20, 22–25 for *CesA* and contigs 1–5, 7–9, 13 for *XTH*, Figure 3)*.* The results also indicated that the sucrose synthase (Susy) and expansin gene families had a lower level of expression in *E. urophylla* compared to the other two species. These results are shown in Figure 3 and Additional file 3: Table S2.

The importance of cell wall genes is highlighted by previously published studies. For example, the cellulose synthase superfamily is responsible for cellulose (*CesA*) and hemicellulose (*Csl*) biosynthesis and deposition [27,28]. The sucrose synthase enzyme catalyzes the reversible conversion of sucrose and UDP to UDP-glucose and fructose [29]. XTH are a large family [30] involved in restructuring primary cell walls to accommodate secondary cell walls [31]. Expansins are cell wall proteins involved in many cell wall modification processes, particularly relaxation and extension, which are essential processes for cell wall enlargement [32]. The action of these proteins results in increased cell wall extensibility and growth [33].

Thus, the expression profile observed here could indicate increased cellulose and hemicelluloses production in *E. globulus,* by gene expression of both precursors of these pathways (by the action of Susy) and final products (*CesA* and *Csl*). Moreover, the expression of XTH and expansins could indicate that the cell wall in this species is intensely modified to accommodate new formed polysaccharides. Since our results also demonstrate the down regulation of these genes in *E. urophylla* in comparison to the other two species, it is plausible to admit that less cellulose and/or hemicelluloses are produced in this species.

#### Phenylpropanoid pathway

Another important metabolic pathway presenting transcriptional differences in the xylems of the three *Eucalyptus* species is the phenylpropanoid pathway, which comprises two branches: lignin biosynthesis and flavonoid biosynthesis. Both pathways share common precursors and enzymes used in the initiation of the metabolism, which determines the level of interdependence among the pathways [34].

We observed the differential expression of at least one member of all gene families present in the phenylpropanoid pathway, except for ferulate 5-hydroxylase (*F5H)* (Additional file 3: Table S2). Due to the importance of phenylpropanoid metabolism in the determination of wood quality, Figure 4 illustrates the two branches of this metabolism and the major enzymes encoded by the genes presenting differential expression among the three *Eucalyptus* species.

The first three enzymes of the phenylpropanoid pathway, phenylalanine ammonium lyase (*PAL*), 4-coumarate-CoA ligase (*4CL*) and cinnamate-4-hydroxylase (*C4H*) are responsible for converting phenylalanine into p-coumaroyl-CoA [35]. For these three enzymes, when all gene members were taken into account, the number of transcripts in *E. grandis* was greater than that of the other two species, whereas this value was lower in *E. globulus*.

The lignin branch includes several gene families encoding the following enzymes: coumaroyl-CoA 3-hydroxylase (*C3H*), cinnamoyl-CoA reductase (*CCR*) and cinnamyl alcohol dehydrogenase (CAD), hydroxycinnamoyl-CoA: quinate shikimate p–hydroxycinnamoyltransferase (*HCT*), caffeoyl-CoA 3-O-methyltransferase (*CCoAOMT*) and caffeic acid: 5-hydroxyferulic acid O-methyltransferase (*COMT*). By analyzing data presented in Figure 4, we observe that the genes in the lignin branch display an overall higher expression in *E. grandis.* In contrast, various genes of the flavonoid branch of the phenylpropanoid pathway, namely those encoding the enzymes chalcone synthase (*CHS*), chalcone isomerase (*CHI*), flavonone 3-hydroxylase (*F3H*) and dihydroflavonol 4-reductase (*DFR*), were expressed at significantly higher levels in *E. urophylla* compared to the two other species (Figure 4)*.*

In general, our results indicated that genes related to lignin biosynthesis are expressed at higher levels in *E. grandis* when compared to *E. globulus* and *E. urophylla*, whereas genes of the flavonoid branch present higher expression in *E. urophylla.* These results illustrate the complexity of the molecular events underlying lignification, since the species that apparently presents a superior lignin content, *E. urophylla*[11], appears to direct the phenylpropanoid pathway towards flavonoids formation.

The flavonoid branch of the phenylpropanoid pathway is known to be up-regulated by various environmental stresses [36], probably due to its protective role in response to a broad variety of stress factors [37]. The up-regulation of this branch in *E. urophylla* may suggest that this species presents more efficient defense mechanisms that enable it to survive in a wide range of environments.

#### Transcription factors

Secondary cell wall formation is regulated by several transcription factors gene families, such as *WRKY*[38,39], *NAC*[40,41], *Myb*[42-44] and *SHN*[45]. Many *Myb* and *WRKY* transcriptions factors were differentially expressed among the xylems of the three species studied here, and in most cases, they presented lower levels of expression in *E. globulus* (Figure 5A and Additional file 3: Table S3). Members of the *Myb* and *WRKY* families are also involved in the regulation of the phenylpropanoid pathway [46,47]. Importantly, one recent work showed that mutations in *WRKY* transcription factors are associated with increased stem biomass [48] and the over-expression of these factors led to improved stress resistance [49]. In this context, it is interesting to observe that some genes encoding transcription factors showed a similar expression profile of genes coding enzymes of the phenylpropanoid pathway (as shown by Figure 5); i.e. these genes were expressed at much higher levels in *E. grandis* and *E. urophylla* in comparison to *E. globulus*.

Another interesting class of transcription factors identified in the *Eucalyptus* xylem transcriptomes is the *AP2/ERF* class. This is a large family of transcription factors that is implicated in the abiotic stress response [50,51]. Many members of this family were expressed in the xylems of the three *Eucalyptus* species and, as observed for the others transcriptions factor discussed above, these genes were down-regulated in *E. globulus* (Figure 5A and Additional file 3: Table S3).

The expression profile of *Myb* and *WRKY* transcription factors, which are involved in the regulation of several developmental process and defense responses, including the regulation of phenylpropanoid pathway [38,43], is probably determinant to the reduction of lignin content in *E. globulus* in comparison to the other species [11]. Also, the reduced expression of *Myb* and *WRKY* genes*,* as well as *AP2/ERF* class, indicates a possible molecular explanation to the poorer adaptability of *E. globulus* in comparison to *E. grandis* and *E. urophylla*: the reduced expression of genes associated with stress and defense responses may result in an inferior ability of overcoming environmental adversities*.*

#### Ubiquitins and heat shock genes

Other relevant results include the differential expression of ubiquitins and heat shock encoding genes (Figure 5B and C, respectively and Additional file 3: Table S3). Many contigs were similar to ubiquitin genes, and among the most strongly expressed genes, there was a tendency of lower expression in *E. globulus* (Figure 5B, members 3, 4, 6, 8, 9, 11–23). Additionally, several members were more highly expressed in *E. urophylla* (members 1, 3, 6, 8, 11, 13, 15, 16, 18–20, 22)*,* which suggests that these species present different physiological responses during growth, development and environmental adaptation.

Ubiquitins are involved in developmental plasticity and environmental adaptation and play major roles in almost all aspects of plant growth and development [52,53]. In addition, these proteins were found to be involved in the regulation of xylem differentiation, thereby allowing cells to initiate and progress through the stages of xylogenesis [17]. Heat shock proteins (HSP) are important in maintaining protein homeostasis inside cells and promoting the proper folding, stability and degradation of polypeptides [54]. In addition, they act as molecular chaperones, regulating protein maturation and the transition between the inactive and active states of signaling molecules, such as receptors, protein kinases and transcriptional regulators [55]. Furthermore, many classes of heat shock proteins are involved in abiotic stress responses [56]. Moreover, previous studies have suggested a role of molecular chaperones in wood formation in *Eucalyptus* and *Pinus*, thereby emphasizing the importance of *hsp* genes as candidate genes for the genetic improvement of wood quality [14,57,58].

Our results indicated the differential expression of several classes of HSP encoding genes among the three *Eucalyptus* species. However, we could observe a clear expression pattern only for the class of small heat shock proteins (smHSP): the expression of smHSP encoding genes was higher in *E. urophylla* and lower in *E. globulus* xylems (Figure 5C and Additional file 3: Table S3).

Because small HSPs appear to be involved in stress tolerance [59], we speculated that *E. urophylla* has a superior ability of overcoming environmental challenges, such as biotic and abiotic stresses. Moreover, these results also support the reduced adaptability to tropical areas that has been observed for *E. globulus*.

#### Disease and stress-resistance genes

Genes related to stress and disease resistance showed a high level of expression in the xylems of *E. globulus* and *E. urophylla* (Figure 6 and Additional file 3: Table S3).

We identified a large class of disease-resistance *R* genes, such as the *CC-NBS-LRR* (*CC*) class and many members of the multidrug resistance (*MDR*) family, among others resistance genes. While *R* genes, such as those of the *CC-NBS-LRR (CC)* class, constitute the key disease-resistance genes in plants [60], the precise role of the large *MDR* gene family has not been well defined yet; however, the association of *MDR* genes with the transport of compounds from the environment, such as herbicides and antibiotics or metabolites, such as flavonoids, has been previously reported, allowing plants to co-exist with toxins [61]. A great number of *CC* genes were highly expressed in the xylem of *E. globulus*, while *MDR* genes showed higher expression levels in the xylem of *E. urophylla*.

Because the genes found here are associated not only to disease-resistance but also with a variety of biotic and abiotic stresses [60,61], it is possible that their expression is also related to the adaptability of these *Eucalyptus* species in areas outside their natural habitat. However, a thorough investigation should be performed to understand the nature of these differences.

Despite a similar expression profile of resistance genes was observed for *E. urophylla* (up-regulation of stress- and disease-related genes), other results related to this species (e.g. up-regulation of phenylpropanoid genes, ubiquitins, transcription factors and smHSPs) are possibly related to a better adaptation to stress conditions in comparison to *E. globulus*. In other words, a system of ‘priming’ resistance may allow *E. urophylla* trees to deal with future stress situations. In contrast, *E. globulus* grows poorly in tropical areas despite the expression of a large number of disease-resistance genes. This result can indicate that this species increases the expression of these genes in an attempt of returning to homeostasis.

This hypothesis could also explain the superior environmental plasticity of *E. urophylla* in comparison to *E. grandis*, which presents an intermediate tolerance when compared to the other two species analyzed here.

In many situations, the expression of ‘resistance genes’ is an attempt to tolerate the stress situation, but not necessarily the plant will be able to resist at first, as we can observe to *E. globulus* plants growth in tropical conditions. These findings highlight the importance of stress tolerance genes in the adaptability of trees from temperate locations to tropical areas.

## Conclusion

The results described here highlight the complexity of the xylem differentiation process, showing that most *Eucalyptus* genes [26] are expressed in developing xylems. Approximately 2400 of the 6518 differentially expressed genes identified in this study are functionally uncharacterized (*no hits*), constituting important candidates for further investigation.

Relevant differences were observed in genes related to construction and/or maintenance of cell wall, which could explain the superior wood quality found in *E. globulus.*

Moreover, our findings suggest that *E. globulus* and *E. urophylla* are subjected to environmental stress, due to a great number of resistance related genes highly expressed in these species. However, the high number of genes regulating or involved in environmental adaptation (*Myb, WRKY, AP2/*ERF, ubiquitins and smHSP) and the distinct regulation of phenylpropanoid genes among these two species may indicate the mechanism by which *E. urophylla* adapts better to these sub-optimal conditions, being prepared to a future stress situation than *E. globulus*, which displays poor growth in non-temperate conditions

In conclusion, our results contribute to the understanding of *Eucalyptus* xylogenesis and expand our knowledge related to the differences in wood quality among different *Eucalyptus* species. Our findings may provide candidate genes for future breeding programs.

## Methods

### Plant materials

Samples of developing xylem tissues of three *Eucalyptus* species (*E. globulus, E. grandis* and *E. urophylla*) were collected (by scraping the tissue after bark removal and immediately frozen in liquid nitrogen and then stored at −80°C) from two three-year-old clones from each species (one sample for mRNA sequencing and one for Real-Time PCR analysis – Additional file 4: Doc file S2). These plants were grown in an experimental area of approximately 100 m^2^ from the International Paper fields in Mogi Guaçu, SP, Brazil (latitude (S): 22°21^′^; longitude (W): 46°58^′^), where the predominant soil is the oxisol, the average annual rainfall is 1278 mm and the average annual temperature is 21.4°C. All samples were collected in April, 30th, 2010 (between 9 am and 11 am).

### RNA extraction

The RNA material was extracted according to the protocol described by Zeng and Yang [62] with modifications proposed by Provost *et al.*[63]. RNA concentration and quality were verified with a Nanodrop 2000 instrument (Thermo Scientific) and confirmed by using a Bioanalyzer Chip DNA 1000 series II (Agilent) and also by agarose gel.

### mRNA sequencing

The mRNA sequencing was performed at the High Throughput Sequencing Facility at the Carolina Center for Genome Sciences (University of North Carolina, USA). For each xylem sample, 10 μg total RNA were used to prepare the mRNAseq library according to the protocol provided by Illumina. The gel extraction step was modified by dissolving excised gel slices at room temperature to avoid the underrepresentation of AT-rich sequences [64]. Library quality control and quantification were performed using a Bioanalyzer Chip DNA 1000 series II (Agilent). For each library, 36 bp single-end sequences were generated in one lane of an Illumina Genome Analyzer II_x_.

### Public data sets

A publically available gene catalog of a commercially grown *E. grandis* X *E. urophylla* hybrid (“urograndis”) was downloaded from *Eucspresso* (http://eucspresso.bi.up.ac.za). The data set consisted of 18,894 contigs longer than 200 bp, and 99.83% of contigs had similarities with the *E. grandis* genome assembly [26]. The *Eucalyptus grandis* genome assembly (Phytozome 8.0) was downloaded from Phytozome (http://www.phytozome.net/eucalyptus).

### Read alignment

The Illumina reads were filtered to exclude ribosomal (using the SILVA database [65]) and low quality reads (phred >= 20). The remaining reads were aligned against the *Eucspresso* contigs using the SOAP2 aligner [66]. To prepare the data for Genebrowser analysis, reads were aligned against the *E. grandis* genome using TopHat [67] to allow spliced alignments. Both programs were configured to allow up two mismatches (SNPs can generate mismatches in the alignment, especially in these cases because the sequences are from different species), discard sequences with ambiguities (Ns) and return all optimal alignments.

### *De novo* assembly

The *de novo* assembly (without a reference) was performed using reads that did not map against the public data set (previously described) using the Trinity assembler [68]; contigs of at least 200 bp were allowed, and the parameter “--run_butterfly” was used.

### Contig annotation

The Autofact program [69] was used to perform an automatic annotation of all *EUCANEXT* contigs. The main feature of Autofact is its capacity of resuming the annotation based on sequence similarity searches in several databases. BLASTx [70] (e-value cutoff of 1e-5) was used to align the contigs against the following public databases: the non-redundant (NR) database of *NCBI*; the Uniref90 and Uniref100database, which contain clustered sets of proteins from Uniprot [71]; the Pfam database of proteins families [72]; the KEGG database of metabolic pathways [73]; and TAIR (version 10 *Arabidopsis* proteins database. Functional annotation (GO) was performed using BLAST2GO [74] with the default parameters.

### Determination of gene expression levels

The expression levels for each gene were estimated using the FPKM (fragments per kilobase of exon per million fragments) value [75]. Three pairwise comparisons (*E. grandis* X *E. globulus*, *E. grandis* X *E. urophylla* and *E. globulus* X *E. urophylla*) between the FPKM values of each species were performed. A differentially expressed gene was defined as having a greater than two-fold difference in FPKM values between species based on a T-test with a 99% confidence rate (cut-off of 0.01).

## Abbreviations

4CL: 4-coumarate-CoA ligase; AP2/ERF: Apetala2/ethylene response factor; BLAST: Basic local alignment search tool; C3H: Coumaroyl-CoA 3-hydroxylase; C4H: Cinnamate-4-hydroxylase; CAD: Cinnamyl alcohol dehydrogenase; CC-NBS-LRR: Coiled-coil –nucleotide-binding site-leucine-rich repeat protein; CCoAOMT: Caffeoyl-CoA 3-O-methyltransferase; CCR: Cinnamoyl-CoA reductase; CesA: Cellulose synthase; Csl: Cellulose synthase-like; CHI: Chalcone isomerase; CHS: Chalcone synthase; COMT: Caffeic acid:5-hydroxyferulic acid O-methyltransferase; DArT: Diversity arrays technology; DRF: Dihydroflavonol 4-reductase; EST: Expressed sequence tag; F3H: Flavonone 3-hydroxylase; F5H: Ferulate 5-hydroxylase; FPKM: Fragments per kilobase of exons per million; GO: Gene ontology; HCT: Hydroxycinnamoyl-CoA: quinate shikimate p–hydroxycinnamoyltransferase; HSP: Heat shock proteins; MDR: Multi drug resistance; Myb: (myeloblastosis) family of transcription factors; NAC: (*NAM, ATAF1, 2* and *CUC2*) family of transcription factors; NCBI: National Center for Biotechnology Information; NR: Non-redundant GenBank database; PAL: Phenylalanine ammonium lyase; RT-qPCR: Real time quantitative polymerase chain reaction; SAGE: Serial analysis of gene expression; SHN: SHINE transcription factor; Susy: Sucrose synthase; WRKY: WRKY aminoacid domain of transcription factor; XTH: Xyloglucan endotransglucosylase/hydrolase.

## Competing interests

The authors declare that they have no competing interests.

## Authors’ contributions

MMS: preparation of the manuscript, sampled the material, prepared the libraries and participated in data analysis; LCN: bioinformatics analysis, construction of EUCANEXT database; ELOC, DCG, JLN, WLM: collaboration in sampling the material, preparation of libraries and data analysis; PJPLT, PM: sequencing of libraries by RNAseq, collaboration in data analysis; JMCM, ACD: coordination of data analysis and revision of manuscript. MFC: coordination of bioinformatics and data analysis; GAGP: coordination of the molecular and bioinformatics analysis and organization of the manuscript. All authors have read and approved the final version of the manuscript.

## Authors’ information

^1^Laboratório de Genômica e Expressão, Departamento de Genética Evolução e Bioagentes, Instituto de Biologia, Universidade Estadual de Campinas, CEP: 13083–970, Campinas, São Paulo, Brasil. ^2^Department of Genetics, School of Medicine, University of North Carolina at Chapel Hill (UNC), Chapel Hill, NC, USA. ^3^Centro de Pesquisa e Desenvolvimento em Recursos Genéticos vegetais, Instituto Agronômico de Campinas, CEP: 13001–970, Campinas, São Paulo, Brasil.

## Supplementary Material

Additional file 1 **FASTA-formatted sequences of the 10,398 contigs formed by the *de novo *assembly.** Click here for file

Additional file 2: Figure S1 Distribution of contig lengths: 29,292 *EUCANEXT* contigs (min: 200, max: 12,053, mean length: 899.5, n50: 1442 bp). **Figure S2.** Distribution tail of FPKM values vs. the contig frequency for each xylem library. Click here for file

Additional file 3: Table S1 All contigs of the EUCANEXT dataset: Contig names, FPKM values, fold-changes and p-values, BLAST results against NR, TAIR and *E. grandis* genome scaffolds. **Table S2.** Cell wall genes: FPKM values, fold-changes and p-values for all genes mentioned in the text. Only isoforms with the most significant expression in the xylem were considered. The dark-gray squares indicate the fold-changes of differentially expressed genes with fold changes>2 and p-values<0.01. The light-gray squares are genes that tended to be differentially expressed, although the fold-change is 2.0>x>1.5 (p-value 0.01)). GL: *E. globulus;* GR: *E. grandis*; UR: *E. urophylla.***Table S3.** Other genes: FPKM values, fold-changes and p-values for all genes mentioned in the text. Only isoforms with the most significant expression in the xylems are considered. The dark-gray squares indicate the fold-changes of differentially expressed genes with fold changes >2 and p-values<0.01. The light-gray squares are genes that tended to be differentially expressed, although the fold-change is 2.0>x>1.5 (p-value 0.01)). GL: *E. globulus*; GR: *E. grandis*; UR: *E. urophylla*. Red, green and brown squares indicate contigs belonging to CC, MDR and other NBS resistance gene families respectively (ZIP 6449 kb). Click here for file

Additional file 4: Doc file S1 Pairwise comparisons. ** Figure S3.** Flow diagram of genes expressed in all xylem libraries (group a, Figure 1). The genes were separated as being “non-differentially expressed” and “differentially expressed”. The differentially expressed genes were analyzed by pairwise comparisons between species. **Figure S4.** GO categories at Biological Process level 3. A: Representative GO categories of genes shared by only two species (groups *b, c* and *d,* Figure 2); B: Representative GO categories of genes expressed in only one species (groups e*, f* and g*,* Figure 2). A and B: The percentage of contigs in each GO category related to the total number of known function contigs is present on the y-axis. **Doc file S2.** Validation by Real Time-PCR (RT-qPCR) [51,76-81]. Click here for file
