# Supplementary figures and images for: Xylem transcription profiles indicate potential metabolic responses for economically relevant characteristics of Eucalyptus species
Source: BMC Genomics. 2013 Mar 22;14:201. doi: 10.1186/1471-2164-14-201 (PMC3618336; doi:10.1186/1471-2164-14-201)

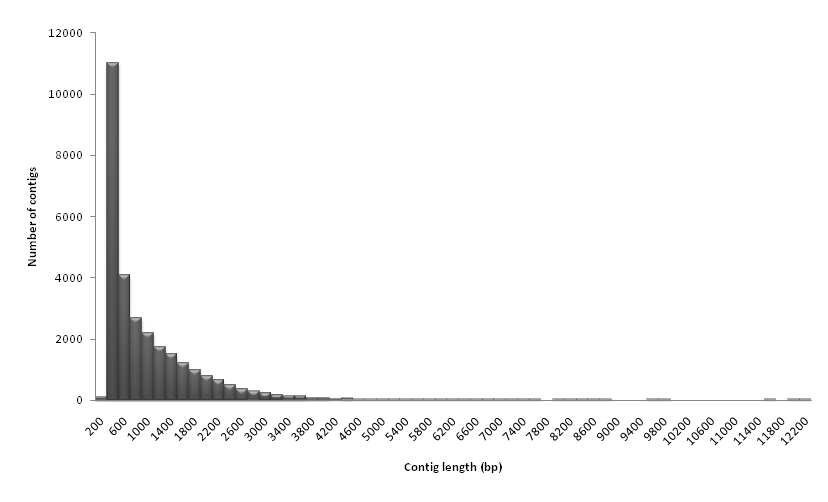

Supplement: Additional file 2: Figure S1 — Distribution of contig lengths: 29,292 EUCANEXT contigs (min: 200, max: 12,053, mean length: 899.5, n50: 1442 bp). Figure S2. Distribution tail of FPKM values vs. the contig frequency for each xylem library. [file 1471-2164-14-201-S2.zip › AdditionalFile2/FigureS1.png]

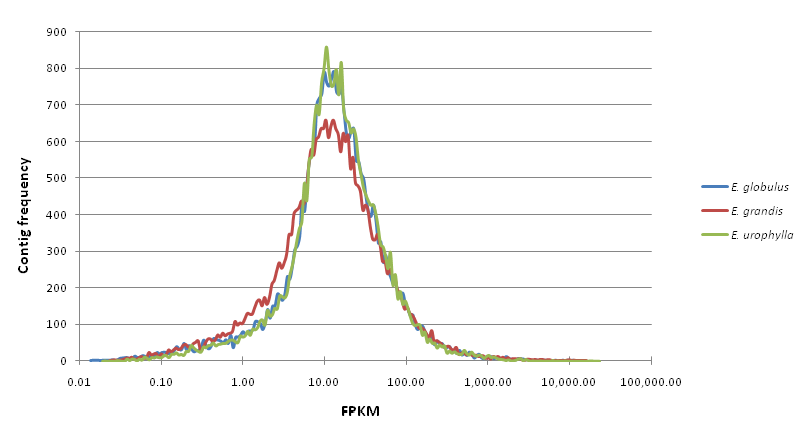

Supplement: Additional file 2: Figure S1 — Distribution of contig lengths: 29,292 EUCANEXT contigs (min: 200, max: 12,053, mean length: 899.5, n50: 1442 bp). Figure S2. Distribution tail of FPKM values vs. the contig frequency for each xylem library. [file 1471-2164-14-201-S2.zip › AdditionalFile2/FigureS2.png]

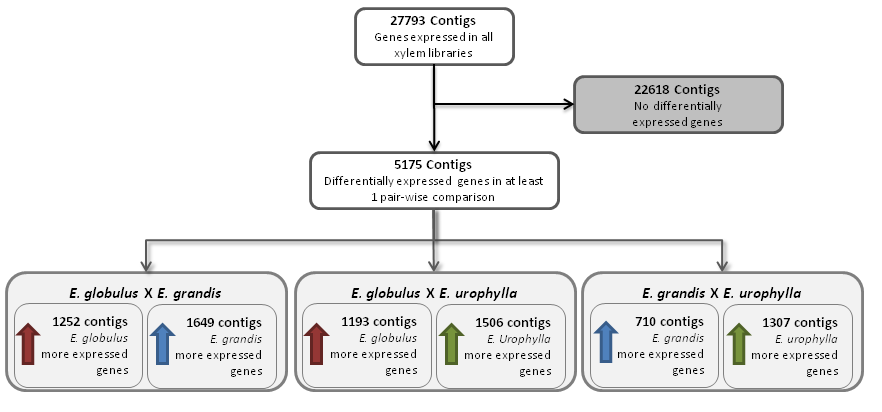

Supplement: Additional file 4: Doc file S1 — Pairwise comparisons. Figure S3. Flow diagram of genes expressed in all xylem libraries (group a, Figure 1). The genes were separated as being “non-differentially expressed” and “differentially expressed”. The differentially expressed genes were analyzed by pairwise comparisons between species. Figure S4. GO categories at Biological Process level 3. A: Representative GO categories of genes shared by only two species (groups b, c and d, Figure 2); B: Representative GO categories of genes expressed in only one species (groups e, f and g, Figure 2). A and B: The percentage of contigs in each GO category related to the total number of known function contigs is present on the y-axis. Doc file S2. Validation by Real Time-PCR (RT-qPCR) [51,76-81]. [file 1471-2164-14-201-S4.zip › AdditionalFile4/FigureS3.png]

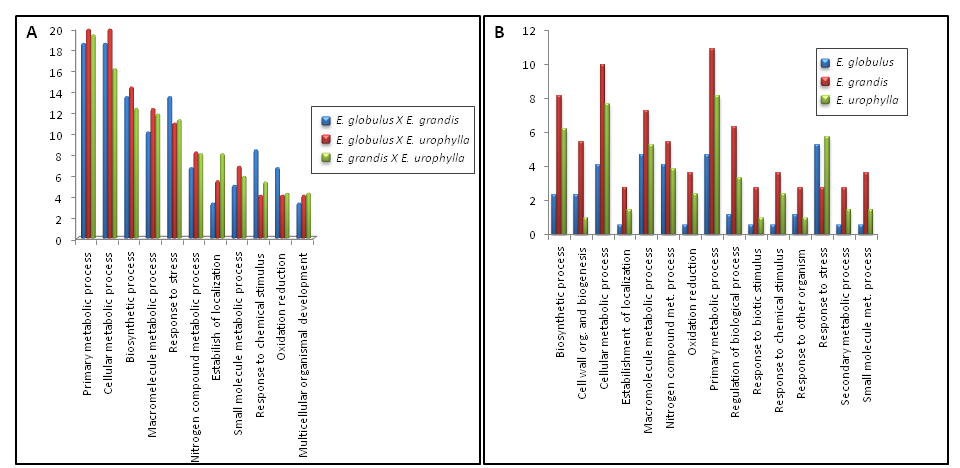

Supplement: Additional file 4: Doc file S1 — Pairwise comparisons. Figure S3. Flow diagram of genes expressed in all xylem libraries (group a, Figure 1). The genes were separated as being “non-differentially expressed” and “differentially expressed”. The differentially expressed genes were analyzed by pairwise comparisons between species. Figure S4. GO categories at Biological Process level 3. A: Representative GO categories of genes shared by only two species (groups b, c and d, Figure 2); B: Representative GO categories of genes expressed in only one species (groups e, f and g, Figure 2). A and B: The percentage of contigs in each GO category related to the total number of known function contigs is present on the y-axis. Doc file S2. Validation by Real Time-PCR (RT-qPCR) [51,76-81]. [file 1471-2164-14-201-S4.zip › AdditionalFile4/FigureS4.png]
