# Supplementary material for: Xylem transcription profiles indicate potential metabolic responses for economically relevant characteristics of Eucalyptus species
Source: BMC Genomics. 2013 Mar 22;14:201. doi: 10.1186/1471-2164-14-201 (PMC3618336; doi:10.1186/1471-2164-14-201)
Supplement: Additional file 4: Doc file S1 — Pairwise comparisons. Figure S3. Flow diagram of genes expressed in all xylem libraries (group a, Figure 1). The genes were separated as being “non-differentially expressed” and “differentially expressed”. The differentially expressed genes were analyzed by pairwise comparisons between species. Figure S4. GO categories at Biological Process level 3. A: Representative GO categories of genes shared by only two species (groups b, c and d, Figure 2); B: Representative GO categories of genes expressed in only one species (groups e, f and g, Figure 2). A and B: The percentage of contigs in each GO category related to the total number of known function contigs is present on the y-axis. Doc file S2. Validation by Real Time-PCR (RT-qPCR) [51,76-81]. [file 1471-2164-14-201-S4.zip › AdditionalFile4/Doc file S1.docx]

**Doc file S1: Pairwise comparisons**

*Contigs with FPKM>0 in all species (Figure2,* group *‘a’)*

A total of 5,175 genes were identified as being differentially expressed in at least one species (Additional File 4, Figure S3). These genes encode ubiquitins, heat shock proteins and transcription factors (MYB, AP2, NAC and WRKY family) in addition to *no hits* (30%), i.e., genes displaying no similarity with any other sequence deposited in the public non-redundant (NR)nucleotide database (NCBI, National Center of Biotechnology Information) and hypothetical proteins (9%). In addition, many of these differentially expressed genes were related to cell wall metabolism (cellulose synthases, UDP-transferases and XETs, for example), nitrogen metabolism (nitrate and nitrite reductases), phenylpropanoid metabolism and resistance to biotic and abiotic stress.

For example, of the 200 genes that were most abundantly expressed (FPKM>504) in all three of the *Eucalyptus* xylems studied here, the majority (72%) constituted functionally characterized genes, 17% encoded hypothetical proteins and 11% were *no hits* genes.

The genes with known functions included many of the traditional housekeeping genes used in RT-qPCR analysis, such as those encoding histone and actin [76], glyceraldehyde-3-phosphate dehydrogenase [77], calmodulins and tubulin [78]. Several other genes were identified as having roles in cell wall construction and plant development, such as members of the cellulose synthase superfamily, sucrose synthase, lignin production-related genes, UDP-glucose dehydrogenase, ubiquitins, chitinases and heat shock proteins.

Among them, many of the traditional housekeeping genes used in RT-qPCR analysis were identified, such as those encoding histone and actin [76], glyceraldehyde-3-phosphate dehydrogenase [77]. This way, these genes can be considered as putative housekeeping genes and could be used as reference genes in RT-qPCR studies and other expression experiments involving the xylems of these three species.

*Contigs with FPKM>0* in *only two species (Figure 2, groups ‘b’, ‘c’ and ‘d’)*

Based on the genes that were expressed only in *E. globulus* and *E. grandis* xylems (Figure 2, group ‘b’), 63% (96 contigs) were not differentially expressed between them. Of the remaining genes, 17 showed higher levels of expression in *E. grandis,* whereas 38 showed higher levels of expression in *E. globulus* (Figure S1.1 A). The majority (61%) constituted *no hits* genes, 29% were functionally characterized and 10% encoded proteins of unknown function (10%). Among the known genes, several were identified as protein kinases and proteins related to stress and pathogen responses.

Among the 335 genes expressed only in the xylems of *E. globulus* and *E. urophylla* (Figure 2, group ‘c’), 104 were considered differentially expressed, 57% constituted *no hits* genes and 5% presented an unknown function. Among the 62 genes that showed higher levels of expression in *E. globulus*, several encoded resistance-related proteins and xyloglucan endotransglycosylases (XET). Additionally, in *E. globulus* xylem, the differential expression of genes encoding a sarcosine oxidase and a nitrate reductase was identified. Many resistance-related genes and ATP phosphoribosyltransferases were included among the 42 genes that showed higher levels of expression in *E. urophylla* (Figure S1.1 B).

Of the 369 contigs present only in *E. grandis* and *E. urophylla* (Figure 2, group ‘d’)*,* 50% constituted *no hits* genes and approximately 6% were genes with unknown functions. Only 87 genes were considered differentially expressed: 40 showed higher levels of expression in *E. grandis* and 47 showed higher levels of expression in *E. urophylla* (Figure S1.1 C). Several of these genes were identified as being related to disease resistance and heat shock proteins.

*Contigs with FPKM>0 in only one species (Figure 2, groups ‘e’, ‘f’ and ‘g’)*

We identified the presence of sarcosine oxidase and expansin genes in *E. globulus,* pyrophosphatase and exportin in *E. grandis,* and ATP phosphoribosyltransferase, inorganic pyrophosphatase and a protein related to cell death in *E. urophylla* (Additional File 3, Table S1).

Another interesting result was the observed up-regulation of a sarcosine oxidase gene in *E. globulus* xylem, as shown for group ‘c’ above.

*Contigs with FPKM=0 in all species* (Figure *2, group ‘h’)*

Among the genes expressed in “urograndis”, but not in the other tree species we found 5 representatives of AP2/ERF transcription factors. These transcription factors appear to regulate the developmental, physiological and biochemical responses of plants under varied environmental conditions, such as drought, high salinity and extreme temperatures (see review in [51]). For the remaining set of 156 genes, most were classified as *no hits* or hypothetical genes. One XET was also identified, although a specific function could not be assigned.

From the analysis of the functional profiles of these groups, it is possible to suggest that stress, disease and pathogen response-related genes presented highly variable levels of expression in the xylem programming of the studied *Eucalyptus* species.


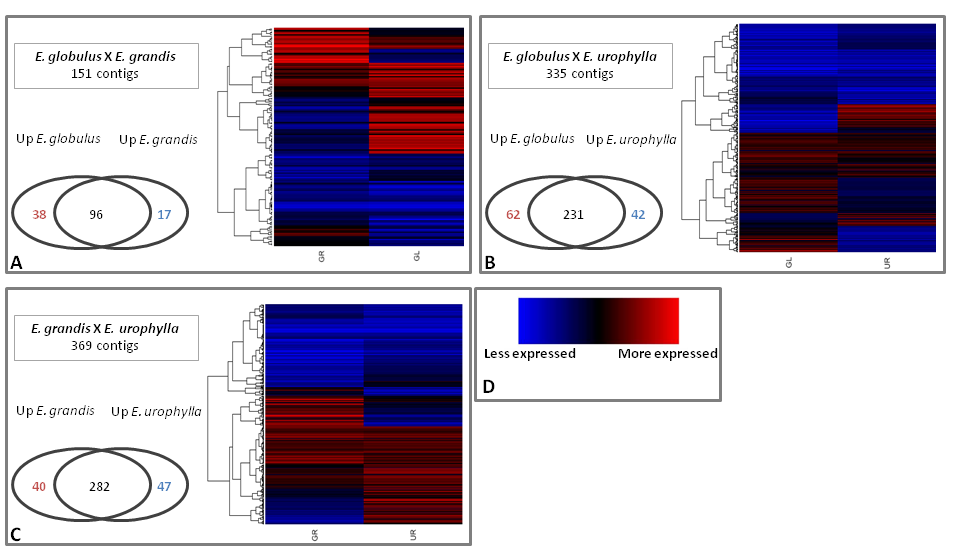


**Figure S1.1**: Heatmaps of gene expression profiles used for pairwise comparisons (groups ‘*b’, ‘c’* and ‘*d*’ in Figure 2) and Venn diagrams showing genes that are differentially and non-differentially expressed among the species. **A**: Gene expression profiling comparison of *E. globulus* and *E. grandis.* **B**: Gene expression profiling comparison of *E. globulus* and *E. urophylla*. **C**: Gene expression profiling comparison of *E. grandis* and *E. urophylla*. **D**: Heatmaps pallet key. GL: *E. globulus,* GR: *E. grandis,* UR: *E. urophylla.*
