# Supplementary material for: Xylem transcription profiles indicate potential metabolic responses for economically relevant characteristics of Eucalyptus species
Source: BMC Genomics. 2013 Mar 22;14:201. doi: 10.1186/1471-2164-14-201 (PMC3618336; doi:10.1186/1471-2164-14-201)
Supplement: Additional file 4: Doc file S1 — Pairwise comparisons. Figure S3. Flow diagram of genes expressed in all xylem libraries (group a, Figure 1). The genes were separated as being “non-differentially expressed” and “differentially expressed”. The differentially expressed genes were analyzed by pairwise comparisons between species. Figure S4. GO categories at Biological Process level 3. A: Representative GO categories of genes shared by only two species (groups b, c and d, Figure 2); B: Representative GO categories of genes expressed in only one species (groups e, f and g, Figure 2). A and B: The percentage of contigs in each GO category related to the total number of known function contigs is present on the y-axis. Doc file S2. Validation by Real Time-PCR (RT-qPCR) [51,76-81]. [file 1471-2164-14-201-S4.zip › AdditionalFile4/Doc file S2.docx]

**Doc file S2: Validation by Real Time-PCR (RT-qPCR)**

**Methods**

The gene expression was evaluated by RT-qPCR for 17 genes randomly selected (according to figure S2, genes were randomly selected to cover major FPKM distribution) and 2 controls using developing xylem samples collected from three year old *E. grandis*, *E. urophylla* and *E. globulus* trees from International Paper fields in Mogi Guaçu, SP, Brazil (Latitude (S): 22^0^21`, Longitude (W): 46^0^58`). As reference condition, we collected a sample from *E. grandis* leaves. All samples were prepared as mentioned in *Methodology* section.

For cDNA synthesis, 1µg of total RNA was treated with RQ1 RNase-Free DNase (Promega). cDNa were synthesized with SuperScript II Reverse Transcriptase (Invitrogen) according to the manufacturer instructions.

RT-qPCR was performed on 96-well plates using the SYBR Green PCR master mix (Applied Biosystems). Non-specific PCR products were identified by the dissociation curves. The relative expression ratio value was calculated according to the Pfaffl equation [79], with the two control genes used for normalization (*SugarT* and *PolyG)* selected from our database.

The pair of primers used for each gene is presented by Table S4.

Table S4: Gene code, gene name and primers sequence used for RT-qPCR validation.

**Results**

Transcript abundance of the 17 genes were estimated and compared with RNAseq results. Gene expression was calculated by fold-change values (Log2) estimated from three pairwise comparisons for each gene: *E. grandis* vs. *E. globulus* (Gr/Gl), *E. grandis* vs. *E*. *urophylla* (Gr/Ur) and *E. globulus* vs. *E. urophylla* (Gl/Ur).

Results were analyzed investigating the relative expression of these genes by RT-qPCR and their respective FPKM values by RNAseq. Table S2.1 presents the results.

Table S2.1: Log2 fold change for RT-qPCR and RNAseq for all three pairwise comparisons.

To evaluate if these genes showed the same trend of expression on collected samples, the expression values were also plotted in a graph and the correlation coefficient (R^2^) was calculated (Figure S2.1), as also presented in previous works [80, 81].


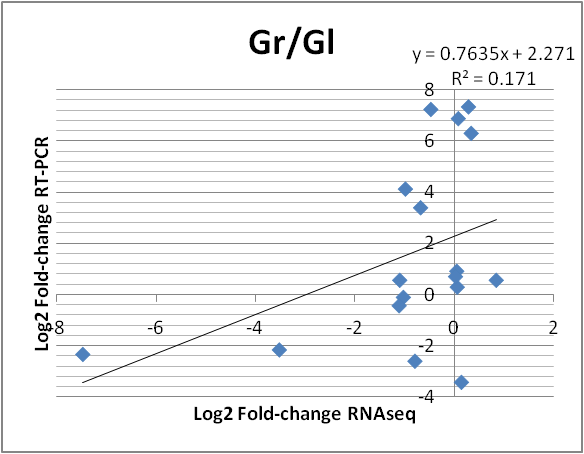

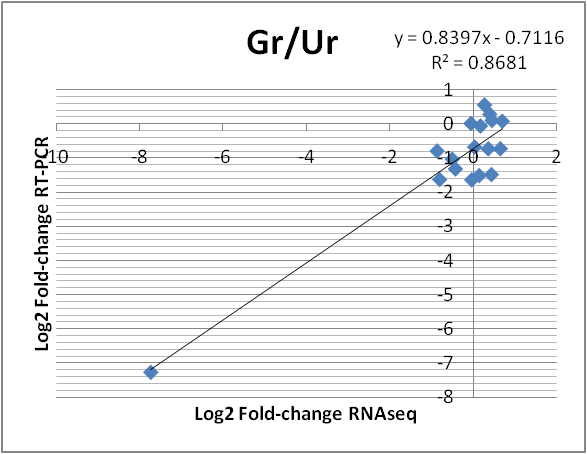

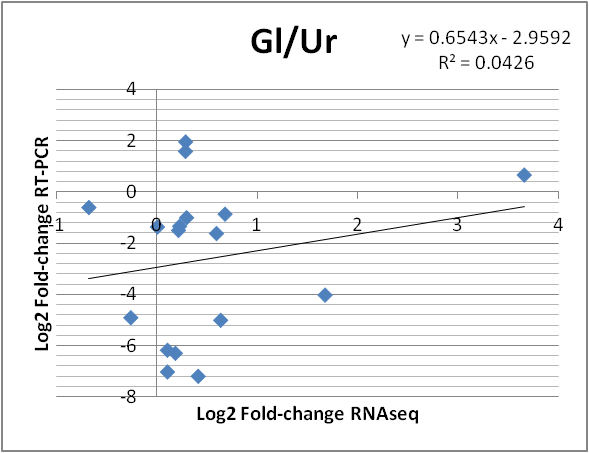


Figure S2.1: Correlation between RT-qPCR and RNAseq for all three pairwise comparisons.

According to results presented by Figure S2.1, the gene expression analyzed by RT-qPCR was strongly correlated to RNAseq values in the comparison between *E. grandis* and *E. urophylla* (R^2^ = 0.87), but much weaker in correlations including *E. globulus (Gr/Gl,* R^2^ = 0.171 *and Gl/Ur,* R^2^ = 0.04). These results may indicate that *E. globulus* have a more heterogeneous molecular profile among distinct trees, while *E. grandis* and *E. urophylla* present more uniform responses.

The heterogeneity of responses may be related to the xylem transcriptional results indicating that *E. globulus* is subjected to environmental stresses (up-regulation of stress- and disease-related genes). Despite same profile was observed for *E. urophylla*, other results in this species (e.g. up-regulation of phenylpropanoid genes) is possibly deviating its phenotype towards a better adaptation to the stress conditions*.* Thus, *E. globulus* trees under stress conditions may be modulating distinctly the transcriptional responses, resulting in a low reproducibility of the samples used for RNAseq and RT-qPCR.

As a general conclusion of this analysis, the correlations between *E. globulus* and other species should be analyzed in a more parsimonious way when using samples collected from stress conditions. Future analysis will greatly improve the comprehension of the phenomenon of variability of expression between plants of *E. globulus*, e.g. due to allelic variation that will be performed soon.
